# Dose and aging effect on patients reported treatment benefit switching from the first overactive bladder therapy with tolterodine ER to fesoterodine: post-hoc analysis from an observational and retrospective study

**DOI:** 10.1186/1471-2490-12-19

**Published:** 2012-07-26

**Authors:** David Castro-Diaz, Pilar Miranda, Francisco Sanchez-Ballester, Isabel Lizarraga, Daniel Arumí, Javier Rejas

**Affiliations:** 1 Department of Urology, Hospital Universitario de Canarias, Santa Cruz de Tenerife, Canarias, Spain; 2 Department of Gynaecology, Hospital de Fuenlabrada, Madrid, Spain; 3 Department of Urology, Hospital General Universitario de Valencia, Valencia, Spain; 4 Medical Unit, Pfizer, S.L.U., Alcobendas (Madrid), Spain; 5 Medical Department, Pfizer Inc. Europe, Alcobendas (Madrid), Spain; 6 Health Economics and Outcomes Research Department, Pfizer, S.L.U, Alcobendas (Madrid), Spain

**Keywords:** Overactive bladder, Fesoterodine, Tolterodine ER, Dose escalation, Age, Patient-reported treatment benefit

## Abstract

**Background:**

Previous randomized studies have demonstrated that fesoterodine significantly improves the Overactive Bladder (OAB) symptoms and their assessment by patients compared with tolterodine extended-release (ER). This study aimed to assess the effect of aging and dose escalation on patient-reported treatment benefit, after changing their first Overactive Bladder (OAB) therapy with tolterodine-ER to fesoterodine in daily clinical practice.

**Methods:**

A post-hoc analysis of data from a retrospective, cross-sectional and observational study was performed in a cohort of 748 OAB adults patients (OAB-V8 score ≥8), who switched to fesoterodine from their first tolterodine-ER-based therapy within the 3–4 months before study visit. Effect of fesoterodine doses (4 mg vs. 8 mg) and patient age (<65 yr vs. ≥65 yr) were assessed. Patient reported treatment benefit [Treatment Benefit Scale (TBS)] and physician assessment of improvement with change [Clinical Global Impression of Improvement subscale (CGI-I)] were recorded. Treatment satisfaction, degree of worry, bother and interference with daily living activities due to urinary symptoms were also assessed.

**Results:**

Improvements were not affected by age. Fesoterodine 8 mg vs. 4 mg provides significant improvements in terms of treatment benefit [TBS 97.1% vs. 88.4%, p < 0.001; CGI-I 95.8% vs. 90.8% p < 0.05)], degree of worry, bother and interference with daily-living activities related to OAB symptoms (p <0.05).

**Conclusions:**

A change from tolterodine ER therapy to fesoterodine with dose escalation to 8 mg in symptomatic OAB patients, seems to be associated with greater improvement in terms of both patient-reported-treatment benefit and clinical global impression of change. Improvement was not affected by age.

## Background

Overactive bladder (OAB) is a lower urinary tract disorder characterized by urgency with or without urge incontinence, often with increased daytime frequency and nocturia [1-4]. The prevalence of OAB increased with age [5-8]. In Spain, the EPICC study showed that the prevalence of OAB, previously estimated in adults ≥40 years of age at 21.5% [9], was 5.9% for women aged 25–64, 4.6% for men aged 50–65 and 38.5% for institutionalized people over 65 [10]. The symptoms associated with OAB can significantly affect the psychological, social, occupational, domestic, and sexual aspects of those who suffer from it [11]. As a result, OAB has a negative impact on the patient’s quality of life [12,13]. Despite the significant impact of OAB on patients’ lives and the availability of treatment options, only a small percentage of elderly patients seek and receive treatment [9,14].

Antimuscarinic agents are the pharmacological mainstay of OAB treatment [15]. Patients, however, often do not respond to them appropriately, largely because of non-compliance, but also due to lack of efficacy or intolerance [16]. In these cases, the symptoms of these patients may be improved by changing the drug [17-20] and behavioural therapy techniques. In this way, previous randomized studies have demonstrated the superior efficacy of fesoterodine over tolterodine [18,19,21].

Therapeutic benefit might be achieved with higher doses. The literature supports that the efficacy of anticholinergics is enhanced by dose escalation [22-24]. Moreover, flexible dosing reflects clinical practice better than fixed dosing [23]. Fesoterodine is a nonselective antimuscarinic agent that has showed a dose-dependent response [22]. This dose response has not been demonstrated with all of the other antimuscarinic agents that offer multiple doses [4].

Several studies have suggested that antimuscarinic agents are generally effective and well-tolerated in older subjects [25-29]. However, the use of drugs in clinical trials markedly differs from that of the routine clinical practice in several aspects, thus limiting the generalization of results [30]. In this sense, non-interventional studies, may provide complementary information on the effectiveness of specific treatments in real clinical practice settings [31]. Then, data from open-label or observational studies exploring the subjective patient perception of the effectiveness of fesoterodine, in symptomatic OAB subjects not satisfied with tolterodine, would also be relevant to clinical management in the daily practice [8,32].

Here, we carried out a post-hoc analysis from the IMPACTA study [32] to evaluate whether aging and dose escalation to fesoterodine 8 mg compared to fesoterodine 4 mg, were associated with higher patient-reported treatment benefit and clinician perception of change, after switching from their first OAB tolterodine ER-based therapy, in daily clinical practice.

## Methods

### Study design and patients

This was a post-hoc analysis of data from a retrospective, cross-sectional observational and multicenter study (IMPACTA), aimed at determining the factors causing treatment change in OAB patients and the resulting degree of satisfaction under normal conditions [32].

Urologists and gynecologists from all over the country were selected at random according to the geographic population density. The original study included outpatients of both genders over 18 years of age, diagnosed with OAB, currently symptomatic in accordance with clinical judgement (OAB-V8 score ≥8) [33]. At the physician’s discretion, treatment was changed due to any cause within the 3–4 months prior to the visit. This post-hoc analysis only included those patients who fulfilled the above-mentioned selection criteria, and who were switched to daily fesoterodine from their first tolterodine ER-based therapy. Fesoterodine dosage could either be maintained at 4 mg or increased to, 8 mg. All patients provided their informed written consent. In accordance with the Spanish recommendations, the study was approved by the Clinical Research Ethics Committee of Hospital General Universitario of Valencia. The study was conducted in accordance with the principles contained in the Declaration of Helsinki for studies in humans.

### Measurements and instruments

The patient-reported treatment benefit of changing was assessed using the self-administered Treatment Benefit Scale (TBS) [34]. The physician-reported treatment improvement resulting from change was assessed by the Clinical Global Impression of Improvement (CGI-I) scale [35]. Patients also completed ad-hoc questions about the self-perceived treatment satisfaction/preference, and degree of worry, bother and interference with daily living activities due to urinary symptoms. Drug compliance was assessed by the Morisky-Green scale [36]. Demographic data, concomitant treatments in the last 3 months and reasons for OAB treatment switch, were also collected by the investigator at the single visit.

The OAB-V8 [33] is a reliable 8-item questionnaire used to identify patients with OAB symptoms. Each item is scored on a 6-point Likert scale ranging from 0 (not at all) to 5 (a very great deal). Total score is obtained by adding up the score of each item. Patients were considered to have OAB if their OAB-V8 score was ≥ 8.

The CGI [35] consists of two subscales. The first subscale, severity of illness *(CGI-S)*, assesses the clinician’s impression of the patient’s current state of illness. It is scored from 1 = normal/not at all ill to 7 = extremely ill. The Global Improvement Subscale *(CGI-I)*, assesses the patient’s improvement or worsening rating 1–7 (1 = very much improved, 2 = much improved, 3 = minimally improved, 4 = no change, 5 = minimally worse, 6 = much worse, 7 = very much worse).

The TBS [34] is a self-administered single-item instrument, used to compare the current state of their urinary problems with their state before the start of the study. It is scored from 1 to 4 (1 = greatly improved, 2 = improved, 3 = no change, 4 = worsened during the treatment).

The Morinsky-Green questionnaire [36] is a four-question survey to assess the patient’s treatment adherence. Patients were classified according to the number of questions answered correctly: compliant (4 questions), partially compliant (3 questions) or non-compliant (≤2 questions).

Ad-hoc *patient questions* were scored using a 5-point Likert-type scale: 1 = not at all, 2 = a little, 3 = some, 4 = quite a bit and 5 = very much/quite a lot. Subjects were asked to rate the degree of worry, bother and impact on their daily-life activities with regard to different OAB symptoms, as well as their satisfaction with current treatment. The preference for current or previous medication was assessed by patients as: *I prefer the new one, I have some preference for the new, I don’t have any preference, I prefer the previous one*. Worry about urinary symptoms included the frequency (increased frequency of micturition during the daytime), incontinence during sexual intercourse, nocturia, frequency of urinary tract infections, urgency, bladder pain, urge incontinence, urinary difficulties (in starting to urinate, resulting in delayed bladder emptying when the subject is ready to urinate) and stress incontinence. The degree of bother was assessed by urinary frequency (the patient believes he/she urinates too often during the day), urgency (sudden, irresistible need to urinate without delay) and urge incontinence (urine loss associated with a strong desire to urinate). Finally, interviewed patients rated the interference of urinary symptoms with their everyday daily-live such as their normal, leisure, occupational and household activities.

### Statistical methodology

Patients were distributed into groups according to doses administered (4 mg, 8 mg) and age (<65 yr, ≥65 yr). As used in several previous studies, [27,28,37] the age range cut-off value to define “older” was set at ≥ 65 years old.

The patient’s degree of improvement or worsening according to the CGI-I subscale was categorized as improved (very much improved, much improved, minimally improved), no change or impaired (minimally worse, much worse, very much worse). The current state of the patient’s urinary problems compared with the state before the start of the study, according to the TBS, was also grouped into three categories: improved (greatly improved, improved) no change or impaired (worsened during the treatment).

A descriptive statistical analysis of all the variables was performed, including central tendency and dispersion measures for continuous variables, and absolute and relative frequencies for categorical variables. Analysis of variance, parametric and robust with Levene test of homogeneity was used to analyze continuous variables and the Chi square test for qualitative variables. Pearson’s correlation coefficient was calculated between ad-hoc OAB questions score and age of patient. ANCOVA test and multivariate analysis controlling for confounding variables (univariate general linear models and logistic regression models) were applied. All results were adjusted for sex, driven treatment change, treatment adherence, treatment length and reason for switching.

The level of significance of the statistical tests was 5% and they were bilateral. All the analyses were performed with the SPSS statistical package, version 17 and 19.

## Results

### Subjects

**Figure 1:**
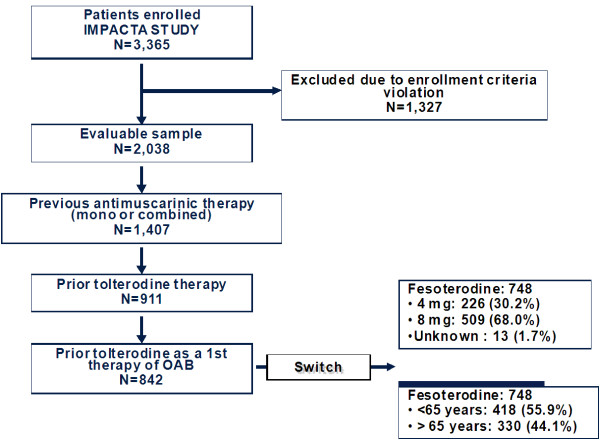
Summary of study populations.

A total of 3,365 patients were included in the IMPACTA study. 748 subjects were analyzed in this post-hoc analysis [55.9% <65 yr, 44.1% ≥65 yr; 30.2% 4 mg, 68.0% 8 mg] from those 842 that received previously tolterodine as a 1^st^ therapy (Figure 1). Table 1 shows demographic and clinical characteristics of the patients. Overall, most subjects were women (83.5%) and the mean age was 61.5 years (Table 1). Patients were of early diagnosis, with a mean time since diagnosis of one year. The principal concomitant conditions, besides high blood pressure, were diabetes mellitus, frequent urinary tract infections and obesity. The majority of the patients were receiving concomitant medications (67.3%). Gender was the only characteristic statistically different between the dose groups (p = 0.014, Table 1). However, the age groups were not homogeneous for gender, body mass index, and mean time since OAB diagnosis. Concomitant conditions, in particular high blood pressure were also more frequent in the older group (Table 1). Fesoterodine 8 mg was prescribed to 69% of the patients under 65 years old and to 70% of the older patients.

**Table 1:** Demographic and clinical characteristics of patients

	**Total N(748)**	**4 mg N(226)**	**8 mg N(509)**	**<65 yr. N(418)**	**≥65 yr. N(330)**
Gender (woman), %	83.5	83.5	73.7^§^	84.9	65.8^*^
Age, mean (SD), yr	61.5(11.0)	61.2(11.1)	61.7(10.8)	53.9(8.1)	71.1(4.9)^*^
BMI, mean (SD), Kg/m^2^	26.6(3.4)	26.5(3.7)	26.6(3.3)	26.0(3.5)	27.3(3.2)^*^
OAB evolution time, mean (SD), d.	389.7(618.6)	405.6(695.7)	383.6(588.1)	316.5(388.6)	482.3(813.7)^*^
OAB-V8 score (0–40)	17.6(7.1)	17.4(7.4)	17.6(7.0)	17.4(7.1)	17.8(7.1)
Concomitant conditions, %		69.8	66.4	59.0	77.9^*^
Obesity (BMI ≥ 30 Kg/m^2^)	12.7	14.6	12.0	10.0	16.1^§^
HBP	45.1	45.1	45.4	34.7	58.2^*^
Urinary tract infections	19.7	18.1	20.0	21.3	17.6^§^
DM	20.6	21.2	19.8	17.5	24.5^§^
Depression	13.4	15.0	12.8	13.4	13.3
CVA	1.2	1.3	1.2	0.2	2.4^§^
Parkinson’s	1.2	1.3	1.0	0.5	2.1
Concomitant medication, %	67.3	69.8	66.4	59.0	77.9^*^

*SD* Standard deviation; *BMI* Body Mass Index; *OAB* (overactive bladder); *HBP* High blood pressure; *DM* Diabetes mellitus; *CVA* Cerebrovascular Accident*p between groups < 0.001; §p between groups < 0.05.

### Switching characteristics

In the majority of cases (75.2%) switching treatment from tolterodine ER to fesoterodine was due to the investigator’s decision (Table 2). The most common reason for switching was lack of effectiveness (66.8%) (Table 2). Between dose groups, this reason was significantly most frequent in the higher dose group (70.9% vs. 58.0%, p < 0.05). Whereas side effects, were the cause of the switch in 23.5% of those titrated to 4 mg and in 16.1% of those escalating to 8 mg (p < 0.05, Table 2).

**Table 2:** Reason for and characteristics of switching

	**Total N(748)**	**4 mg N(226)**	**8 mg N(509)**	**<65 yr. N(418)**	**≥65 yr. N(330)**
Driven treatment change, %					
Patient request	24.8	29.8	21.7	24.7	24.9
Investigator decision	75.2	70.2	78.3^§^	75.3	75.1
Principal reason for switching, %					
Lack of effectiveness	66.8	58.0	70.9^§^	67.0	66.7
Side-effects	18.4	23.5	16.1	17.0	20.3
Bad compliance	6.1	8	5.3	7.2	4.8
Others	8.5	10.5	7.7	8.9	8.2
Treatment length^‡^, mean (SD), d.	66.2(36.2)	60.1(34.8)	68.8(36.7)^§^	63.8(36.1)	69.2(36.2)^§^
Treatment compliance ^£^, %	31.0	24.9	33.5§	31.6	30.3

‡at the time of study visit; £:% of compliers (4 Morinsky-Green questions answered correctly); *:p between groups < 0.001; §:p between groups < 0.05.

When patients were evaluated in this trial, subjects had been on treatment with fesoterodine for nearly two months (Table 2). The treatment length was significantly higher in the greater dose group (68.8 vs. 60.1 days; p < 0.003) and in the older patients (69.2 vs. 63.8 days; p < 0.042). Thirty-one percent of the patients showed compliance with the current treatment (correct response to 4 out of the 4 questions in the Morinsky-Green questionnaire). Compliance rate was higher with 8 mg dosing (33.5% vs. 24.9%, p = 0.035) and similar between age groups (Table 2).

### Treatment benefit of change

The illness status of the patients after the change of treatment was mildly ill, CGI-S mean (SD) [total 3.2 (1.2); doses groups (3.2 (1.1) 4 mg vs. 3.1 (1.2) 8 mg, p = 0.252); age groups 3.1 (1.2) <65 yr vs. 3.2 (1.1) ≥65 yr, p = 0.142)].

**Figure 2:**
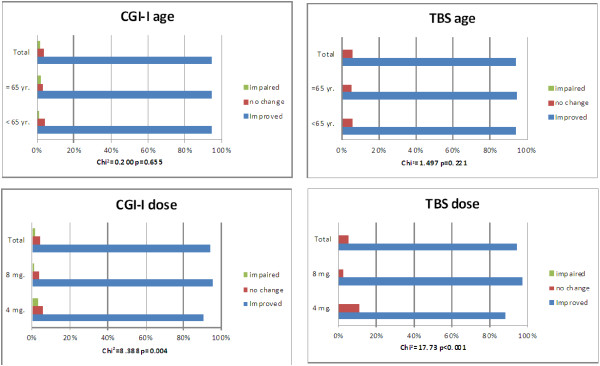
**Patient and physician-reported treatment benefit after switching from tolterodine ER to fesoterodine.** CGI-I, Global Improvement subscale; TBS, Treatment Benefit Scale.

Patient and physician-reported treatment benefit of changing is showed in Figure 2. 94.4% of the overall patients improved in the physician’s judgement as per the CGI-I scoring after the change to fesoterodine. 94.2% of the patient rating improved on the TBS (Figure 2). The differences between age groups were not statistically significant (p = 0.655, Figure 2). Regarding dose groups, the physician-reported treatment benefit (CGI-I) was significantly higher with the 8 mg dose (95.8% vs. 90.8%; p = 0.04). The proportion of patients who reported being improved, according to TBS, was also significantly higher in the fesoterodine 8 mg group compared with 4 mg (97.1% vs. 88.4%; p < 0.001). Table 3 and 4 summarize patients urinary symptoms scoring in relation to bother, worry and impact on daily living items by age and dose, respectively. The symptoms that caused most concern (somewhat/quite a bit) to the patients as a whole were *increased frequency during the day*, *urgency*, *nocturia* and *urge incontinence* (Table 3). The *strong desire to urinate* was the symptom that causes greatest bother. OAB symptoms interfered principally with *leisure* and *usual activities*.

**Table 3:** Urinary symptoms improvement after switching from tolterodine ER to fesoterodine according to age of patients

**Urinary symptoms** **(not at all = 0 to quite a lot = 5)**	**Total**	**<65 years**	**>65 years**	**P value between groups**	**r**^**₤**^
*Worry*					
Frequency	3.2 (3.1-3.3)	3.2 (3.1-3.3)	3.3 (3.1-3.4)	0.325	0.056
Incontinence during sexual attempt	2.2 (2.2-2.3)	2.3 (2.2-2.4)	2.0 (1.9-2.2)	0.008	−0.128^‡^
Nocturia	3.0 (2.9-3.1)	3.0 (2.9-3.1)	3.1 (3.0-3.3)	0.051	0.104^*^
Frequency of infections	2.4 (2.3-2.5)	2.4 (2.2-2.5)	2.4 (2.2-2.5)	0.923	0.022
Urgency	3.2 (3.1-3.3)	3.2 (3.1-3.3)	3.3 (3.1-3.4)	0.585	0.044
Bladder pain	2.3 (2.3-2.4)	2.3 (2.2-2.4)	2.4(2.2-2.5)	0.775	0.034
Urge incontinence	3.1 (3.0-3.3)	3.1 (2.9-3.2)	3.2 (3.1-3.3)	0.211	0.055
Urinary difficulties	2.2 (2.1-2.3)	2.1 (2.0-2.2)	2.3 (2.2-2.4)	0.070	0.141^‡^
Stress incontinence	2.2 (2.1-2.3)	2.1 (2.0-2.2)	2.2 (2.1-2.4)	0.266	0.028
*Bother*					
Urinary frequency	3.2 (3.2-3.3)	3.2 (3.1-3.3)	3.3 (3.2-3.4)	0.453	0.029
Strong desire to urinate	3.3 (3.2-3.4)	3.3 (3.1-3.4)	3.3 (3.2-3.4)	0.642	0.022
Urine loss associated with a strong desire to urinate	3.1 (3.0-3.2)	3.1 (2.9-3.2)	3.2 (3.1-3.3)	0.117	0.055
*Interference with daily-living activities*					
Usual activities	3.0 (2.9-3.1)	3.0 (2.9-3.1)	3.1 (2.9-3.2)	0.519	0.034
Leisure	3.1 (3.0-3.2)	3.1 (3.0-3.2)	3.1 (3.0-3.2)	0.761	−0.005
Work/domestic activities	2.5 (2.4-2.6)	2.7 (2.6-2.8)	2.2 (2.0-2.3)	<0.001	−0.197^‡^

p significance level adjusted by sex, driven of treatment change, treatment adherence, treatment length, reason for switching; Values are mean (95% Confidence Interval) ^₤^ Pearson coefficient of correlation between symptom score and age. *p < 0.05; ‡p ≤ 0.001.

**Table 4:** Urinary symptoms improvement after switching from tolterodine ER to fesoterodine according to fesoterodine dose at the study visit

**Urinary symptom** **(not at all = 0 to quite a lot = 5)**	**Total**	**4 mg**	**8 mg**	**p value between groups**
*Worry*				
Frequency	3.2 (3.1-3.3)	3.4 (3.3-3.5)	3.1 (3.0-3.2)	0.001
Incontinence during sexual attempt	2.2 (2.2-2.3)	2.1 (1.9-2.2)	2.2 (2.1-2.4)	0.075
Nocturia	3.0 (2.9-3.1)	3.2 (3.1-3.4)	3.0 (2.9-3.1)	0.010
Frequency of infections	2.4 (2.3-2.5)	2.4 (2.2-2.5)	2.4 (2.3-2.5)	0.812
Urgency	3.2 (3.1-3.3)	3.4 (3.2-3.5)	3.2 (3.1-3.3)	0.026
Bladder pain	2.3 (2.3-2.4)	2.4 (2.3-2.6)	2.3 (2.2-2.4)	0.207
Urge incontinence	3.1 (3.0-3.3)	3.3 (3.2-3.5)	3.1 (3.0-3.2)	0.008
Urinary difficulties	2.2 (2.1-2.3)	2.2 (2.0-2.3)	2.2 (2.1-2.3)	0.691
Stress incontinence	2.2 (2.1-2.3)	2.1 (1.9-2.3)	2.2 (2.1-2.3)	0.304
*Bother*				
Urinary frequency	3.2 (3.2-3.3)	3.4 (3.3-3.6)	3.2 (3.1-3.2)	0.001
Strong desire to urinate	3.3 (3.2-3.4)	3.5 (3.4-3.7)	3.2 (3.1-3.3)	<0.001
Urine loss associated with a strong desire to urinate	3.1 (3.0-3.2)	3.3 (3.1-3.5)	3.0 (2.9-3.1)	0.007
*Interference with daily-living activities*				
Usual activities	3.0 (2.9-3.1)	3.1 (3.0-3.3)	3.0 (2.9-3.1)	0.119
Leisure	3.1 (3.0-3.2)	3.2 (3.1-3.4)	3.0 (2.9-3.1)	0.032
Work/domestic activities	2.5 (2.4-2.6)	2.5 (2.3-2.6)	2.4 (2.3-2.5)	0.717

p significance level adjusted by sex, driven of treatment change, treatment adherence, treatment length, reason for switching; Values are mean (95% Confidence Interval).

Compared with younger subjects, patients ≥ 65 showed similar results except for *worry about incontinence during sexual attempt* (p = 0.008) and *interference with work/domestic activities* (p < 0.001) which were significantly higher in the younger group (Table 3).

Regarding dose groups, patients who received a dose escalation to 8 mg, rated significantly lower their worry and bother-related to all usual OAB symptoms as *frequency*, *nocturia*, *urgency* and *urge incontinence,* compared with 4 mg. (p < 0.05, Table 4). The interference with daily-living activities due to OAB symptoms, improved with higher dosing, particularly in the case of leisure (p < 0.05, Table 4).

Overall, patients reported being satisfied (*somewhat/quite a bit*) with fesoterodine treatment, with a mean score (SD) of 3.7 (0.9). Nearly all subjects (92.8%) declared *preferring/having some preference* for fesoterodine.

Younger and older patients both showed a similar treatment satisfaction [mean (SD): 3.7 (0.9) vs. 3.6 (0.8), p = 0.256] and preference for the new treatment (mean (SD): 93.0% vs. 92.4%, p = 0.888).

Between dose groups, patient satisfaction with the new treatment was greater with the higher dosing [mean (95% CI): 3.7 (3.7-3.8) vs. 3.5 (3.4-3.6), p = 0.003]. The proportion of patients who reported *preferring/having some preference* for fesoterodine was also significantly higher with the dose of 8 mg (94.8% vs. 88.0%; p = 0.001).

## Discussion

This was a post-hoc analysis, from a non-interventional study, involving patients who expressed dissatisfaction with their prior and first OAB treatment with tolterodine ER, principally because of lack of effectiveness or tolerability problems.

The present data, show that switching to flexible doses of fesoterodine 4 or 8 mg, in the usual clinical practice, provided improvements in the state of the urinary problems in a very high portion of patients, both in the physician’s and patient’s point of view. Furthermore, patients declared being satisfied with the new treatment, and more than 90% of them, reported preferring fesoterodine treatment instead of tolterodine ER. Our findings are in consonance with those reported in an open-label study [8] in which flexible-dose fesoterodine significantly improved OAB symptoms and rates of treatment satisfaction in subjects who were dissatisfied with prior tolterodine therapy.

One possible reason for such findings may lie in the pharmacological profile of fesoterodine. In contrast to tolterodine, which is metabolized in the liver via cytochrome P450 (CYP) 2D6 to produce the active metabolite 5-hydroxymethyl tolterodine (5-HMT), hepatic enzymes are not involved in the conversion of fesoterodine to 5-HMT [19,22]. Fesoterodine is extensively and rapidly converted to 5-HMT, so no fraction of fesoterodine is detectable in plasma after administration, unlike tolterodine [19,22]. Moreover, there is substantial interindividual variability in CYP2D6 metabolic activity, while, the esterases that convert fesoterodine to 5-HMT do not exhibit genotypic variations [8,38,39]. Thus, the pharmacokinetic variability among individuals treated with fesoterodine is lower [8].

Additional explanations for these findings, particularly the high figures of effectiveness as perceived by both clinicians and patients, could go in two ways. In one way, previous randomized studies have demonstrated the superior efficacy of fesoterodine over tolterodine [18,19,21]. Therefore, it should be no surprise to see replication of such results in routine medical practice. On the other hand, these findings should also be interpreted in the light of the fact that the cohort of patients included in this analysis needed a change in its previous tolterodine-based therapy of their OAB symptoms. Moderate improvements, as shown in the urinary questions (see Tables 3 and 4), could then be perceived as important benefits from the patient’s or clinician’s perspective, because such moderate improvements were observed in the urinary symptoms that best define the OAB condition. Finally, no possible ceiling effect in the CGI-I scale has been described yet. However, as the TBS is a one-item scale with four possible categories of response (greatly improved, improved, no changed and worsened during the treatment), a ceiling effect could not be completely ruled-out.

To date, few studies have addressed the efficacy and tolerability of fesoterodine in elderly patients [29,40]. Here, the benefits of switching to fesoterodine do not seem to be age-related. Thus, the percentage of younger patients who reached treatment benefit (according to TBS and CGI-I scales) was similar to that in older subjects. No statistically significant difference was observed regarding the degree of worry, bother or interference with the daily-living activities due to usual urinary symptoms, between younger patients and those over 65 years. Our results were compatible with those from randomized studies with antimuscarinic agents for OAB, which showed a similar efficacy irrespective of age [27,40]. Conversely, these results differ from those obtained in a post-hoc analysis of data from two randomized fesoterodine 4 mg and 8 mg studies. There, patients stratified according to age into three categories (<65, ≥65- < 75, ≥75), showed greater treatment response and improvements in the two younger groups, while, in the ≥75 years group, improvement was only observed with the 8 mg dose [29].

The results presented here show that compared with 4 mg, fesoterodine dose escalation to 8 mg provides significant additional improvements. In the patient’s opinion, worry, bother and interference–related OAB symptoms, improved or showed a trend to greater improvement with higher dosing. 8 mg of fesoterodine provides also higher improvement in term of both patient-reported-treatment benefit and clinical global impression of change.

In addition, compared with subjects receiving 4 mg, a significantly greater proportion of subjects receiving fesoterodine 8 mg, reported drug compliance preference and satisfaction with the new treatment. Our findings, stemming from the usual clinical practice, were in consonance with those reported in previous randomized studies, where the higher 8-mg dose provides additional benefit compared with the lower dose at most end points [22,41]. Up to now, we are unaware of the existence of non-interventional studies that addressed the issue of efficacy of fesoterodine dose-escalation. The only open-label, single-arm study that addressed it, did not compare the efficacy between subjects treated with 4-mg dose throughout study and subjects who escalated to the 8-mg dose [8].

The availability of two doses of fesoterodine allows for individualization of patient care [8]. Dose escalation may allow for improved outcomes in those patients who reported good tolerability and desire greater symptom relief [8]. In the above-mentioned open-label study, half of the subjects opted to escalate their fesoterodine 4 mg dose to 8 mg [8]. In the present study nearly 70% of subjects received the higher fesoterodine 8 mg dose. Here, the reasons for dose escalation were not recorded either. However, this could be indicative of the fact that, the percentage of patients treated with 8 mg that switched due to lack of tolterodine effectiveness, was significantly higher than those in the 4 mg group. While, side effects were the cause for switching in 23.5% of those titrated to 4 mg vs. 16.1% of those escalating to 8 mg.

The present post-hoc analysis had limitations. We did not capture reasons why physicians/patients did or did not opt for dose escalation. This may be important, because the reasons underlying the decision whether or not to increase the dose, probably varied between individuals, but reflect optimization of the balance between efficacy and tolerability [8]. Additionally, there were limitations inherent in the observational design of the study. In this study, the groups of patients were not well-balanced. Most of the patient characteristics assessed were different between groups. One of them is the treatment length. This could be a confounding factor, but it is not, because the mean time of treatment length was more than two months in all our groups, and significant fesoterodine improvements had been reported as early as two weeks after initiation of treatment [22].

In spite of the limitations of the study, it showed the benefit that patients dissatisfied with tolterodine ER perceive upon changing to fesoterodine, which is greater at higher doses. Patient perception of OAB treatment outcomes may be a useful indicator of benefit and might help drive persistence on treatment, which is known to be poor in OAB [20].

## Conclusions

Our analysis suggests that a change from tolterodine ER-based therapy to fesoterodine in symptomatic OAB patients was associated with increased patient treatment benefit. Improvement was not affected by age. Moreover, fesoterodine dose escalation to 8 mg provided significant additional improvement in terms of treatment response, treatment satisfaction, degree of worry-related urinary symptoms, and drug compliance.

## Competing interests

This study was funded by Pfizer, S.L.U. IL and JR are employees of Pfizer, S.L.U. and DA is an employee of Pfizer Inc, Europe. DCD, PM and FSB have not received any financial support from Pfizer for writing or interpreting the present research, and declare that they do not have any conflict of interests as a consequence of this paper. A funding paid by Pfizer was received by Esther Tapia for drafting the manuscript.

## Authors’ contributions

DCD, PM, FSB, IL and DA participated in the original idea and design of the study, interpreted results of analysis and actively reviewed and critiqued the manuscript for important intellectual content. JR participated in the analysis and interpretation of data. All authors read and approved the final manuscript.

## Pre-publication history

The pre-publication history for this paper can be accessed here:

http://www.biomedcentral.com/1471-2490/12/19/prepub

## References

[B1] Abrams P Cardozo L Fall M Griffiths D Rosier P Ulmsten U Van KP Victor A Wein A The standardisation of terminology of lower urinary tract function: report from the Standardisation Sub-committee of the International Continence Society Neurourol Urodyn 2002 21 167 178 10.1002/nau.10052 11857671

[B2] Abrams P Artibani W Cardozo L Dmochowski R Van KP Sand P Reviewing the ICS 2002 terminology report: the ongoing debate Neurourol Urodyn 2009 28 287 10.1002/nau.20737 19350662

[B3] Wein AJ Rovner ES Definition and epidemiology of overactive bladder Urology 2002 60 7 12 12493342 10.1016/s0090-4295(02)01784-3

[B4] Ellsworth P Fesoterodine for the treatment of urinary incontinence and overactive bladder Ther Clin Risk Manag 2009 5 869 876 19956551 10.2147/tcrm.s6483 PMC2781061

[B5] Stewart WF Van Rooyen JB Cundiff GW Abrams P Herzog AR Corey R Hunt TL Wein AJ Prevalence and burden of overactive bladder in the United States World J Urol 2003 20 327 336 12811491 10.1007/s00345-002-0301-4

[B6] Irwin DE Milsom I Hunskaar S Reilly K Kopp Z Herschorn S Coyne K Kelleher C Hampel C Artibani W Abrams P Population-based survey of urinary incontinence, overactive bladder, and other lower urinary tract symptoms in five countries: results of the EPIC study Eur Urol 2006 50 1306 1314 10.1016/j.eururo.2006.09.019 17049716

[B7] Milsom I Abrams P Cardozo L Roberts RG Thuroff J Wein AJ How widespread are the symptoms of an overactive bladder and how are they managed? A population-based prevalence study BJU Int 2001 87 760 766 11412210 10.1046/j.1464-410x.2001.02228.x

[B8] Wyndaele JJ Goldfischer ER Morrow JD Gong J Tseng LJ Guan Z Choo MS Effects of flexible-dose fesoterodine on overactive bladder symptoms and treatment satisfaction: an open-label study Int J Clin Pract 2009 63 560 567 10.1111/j.1742-1241.2009.02035.x 19348029 PMC2705818

[B9] Castro D Espuna M Prieto M Badia X Prevalence of overactive bladder in Spain: a population-based study Arch Esp Urol 2005 58 131 138 15847270 10.4321/s0004-06142005000200006

[B10] Martinez AE Ruiz Cerda JL Gomez PL Ramirez BM Delgado OF Rebollo P Gonzalez-Segura AD Arumi D [Prevalence of urinary incontinence and hyperactive bladder in the Spanish population: results of the EPICC study] Actas Urol Esp 2009 33 159 166 19418840 10.1016/s0210-4806(09)74117-8

[B11] Abrams P Kelleher CJ Kerr LA Rogers RG Overactive bladder significantly affects quality of life Am J Manag Care 2000 6 S580 S590 11183901

[B12] Liberman JN Hunt TL Stewart WF Wein A Zhou Z Herzog AR Lipton RB Diokno AC Health-related quality of life among adults with symptoms of overactive bladder: results from a U.S. community-based survey Urology 2001 57 1044 1050 10.1016/S0090-4295(01)00986-4 11377301

[B13] Coyne KS Payne C Bhattacharyya SK Revicki DA Thompson C Corey R Hunt TL The impact of urinary urgency and frequency on health-related quality of life in overactive bladder: results from a national community survey Value Health 2004 7 455 463 10.1111/j.1524-4733.2004.74008.x 15449637

[B14] Patel B Bavendam T Badlani G Use of antimuscarinics in the elderly ScientificWorldJournal 2009 9 459 465 19526185 10.1100/tsw.2009.55 PMC5823097

[B15] Ouslander JG Management of overactive bladder N Engl J Med 2004 350 786 799 10.1056/NEJMra032662 14973214

[B16] D'Souza AO Smith MJ Miller LA Doyle J Ariely R Persistence, adherence, and switch rates among extended-release and immediate-release overactive bladder medications in a regional managed care plan J Manag Care Pharm 2008 14 291 301 18439051 10.18553/jmcp.2008.14.3.291 PMC10438114

[B17] Swift SE Siami P Forero-Schwanhaeuser S Diary and patient-reported outcomes in patients with severe overactive bladder switching from tolterodine extended release 4 mg/day to solifenacin treatment: an open-label, flexible-dosing, multicentre study Clin Drug Investig 2009 29 305 316 10.2165/00044011-200929050-00003 19366272

[B18] Kaplan SA Schneider T Foote JE Guan Z Carlsson M Gong J Superior efficacy of fesoterodine over tolterodine extended release with rapid onset: a prospective, head-to-head, placebo-controlled trial BJU Int 2011 107 1432 1440 10.1111/j.1464-410X.2010.09640.x 20860717

[B19] Herschorn S Swift S Guan Z Carlsson M Morrow JD Brodsky M Gong J Comparison of fesoterodine and tolterodine extended release for the treatment of overactive bladder: a head-to-head placebo-controlled trial BJU Int 2010 105 58 66 10.1111/j.1464-410X.2009.09086.x 20132103

[B20] Zinner N Kobashi KC Ebinger U Viegas A Egermark M Quebe-Fehling E Koochaki P Darifenacin treatment for overactive bladder in patients who expressed dissatisfaction with prior extended-release antimuscarinic therapy Int J Clin Pract 2008 62 1664 1674 10.1111/j.1742-1241.2008.01893.x 18811599 PMC2680263

[B21] Chapple CR Van Kerrebroeck PE Junemann KP Wang JT Brodsky M Comparison of fesoterodine and tolterodine in patients with overactive bladder BJU Int 2008 102 1128 1132 10.1111/j.1464-410X.2008.07907.x 18647298

[B22] Khullar V Rovner ES Dmochowski R Nitti V Wang J Guan Z Fesoterodine dose response in subjects with overactive bladder syndrome Urology 2008 71 839 843 10.1016/j.urology.2007.12.017 18342923

[B23] Dmochowski RR Peters KM Morrow JD Guan Z Gong J Sun F Siami P Staskin DR Randomized, double-blind, placebo-controlled trial of flexible-dose fesoterodine in subjects with overactive bladder Urology 2010 75 62 68 10.1016/j.urology.2009.09.018 19931895

[B24] MacDiarmid SA Overactive bladder: improving the efficacy of anticholinergics by dose escalation Curr Urol Rep 2003 4 446 451 10.1007/s11934-003-0025-z 14622497

[B25] Malone-Lee JG Walsh JB Maugourd MF Tolterodine: a safe and effective treatment for older patients with overactive bladder J Am Geriatr Soc 2001 49 700 705 10.1046/j.1532-5415.2001.49144.x 11454106

[B26] Michel MC Schneider T Krege S Goepel M Does gender or age affect the efficacy and safety of tolterodine? J Urol 2002 168 1027 1031 10.1016/S0022-5347(05)64567-3 12187215

[B27] Zinner NR Mattiasson A Stanton SL Efficacy, safety, and tolerability of extended-release once-daily tolterodine treatment for overactive bladder in older versus younger patients J Am Geriatr Soc 2002 50 799 807 10.1046/j.1532-5415.2002.50203.x 12028164

[B28] Chapple C DuBeau C Ebinger U Rekeda L Viegas A Darifenacin treatment of patients > or = 65 years with overactive bladder: results of a randomized, controlled, 12-week trial Curr Med Res Opin 2007 23 2347 2358 10.1185/03007X226294 17706004

[B29] Kraus SR Ruiz-Cerda JL Martire D Wang JT Wagg AS Efficacy and tolerability of fesoterodine in older and younger subjects with overactive bladder Urology 2010 76 1350 1357 10.1016/j.urology.2010.03.097 20974482

[B30] Stolley PD Laporte J Strom BL The Public Health, the University, and Pharmacoepidemiology Pharmacoepidemiology 2000 Third John Wiley & Sons

[B31] Gallagher RM Naturalistic study designs in samples with refractory pain: advantages and limitations Pain Med 2004 5 143 145 10.1111/j.1526-4637.2004.04036.x 15209966

[B32] Castro D Miranda P Sanchez-Ballester F Arumi D Lizarraga I Ebel C Assessment of reasons for overactive bladder treatment change Actas Urol Esp 2011 35 73 79 10.1016/S2173-5786(11)70022-1 21296454

[B33] Coyne KS Zyczynski T Margolis MK Elinoff V Roberts RG Validation of an overactive bladder awareness tool for use in primary care settings Adv Ther 2005 22 381 394 10.1007/BF02850085 16418145

[B34] Colman S Chapple C Nitti V Haag-Molkenteller C Hastedt C Massow U Validation of treatment benefit scale for assessing subjective outcomes in treatment of overactive bladder Urology 2008 72 803 807 10.1016/j.urology.2008.05.033 18722655

[B35] Guy W Clinical Global Impressions: In ECDEU Assessment Manual for Psychopharmacology. Revised DHEW Pub. (ADM) 1976 Rockville, MDL: National Institute for Mental Health 218 222

[B36] Morisky DE Green LW Levine DM Concurrent and predictive validity of a self-reported measure of medication adherence Med Care 1986 24 67 74 10.1097/00005650-198601000-00007 3945130

[B37] Wagg A Wyndaele JJ Sieber P Efficacy and tolerability of solifenacin in elderly subjects with overactive bladder syndrome: a pooled analysis Am J Geriatr Pharmacother 2006 4 14 24 10.1016/j.amjopharm.2006.03.004 16730617

[B38] Brynne N Dalen P Alvan G Bertilsson L Gabrielsson J Influence of CYP2D6 polymorphism on the pharmacokinetics and pharmacodynamic of tolterodine Clin Pharmacol Ther 1998 63 529 539 10.1016/S0009-9236(98)90104-7 9630826

[B39] Malhotra BK Wood N Sachse R Influence of age, gender, and race on pharmacokinetics, pharmacodynamics, and safety of fesoterodine Int J Clin Pharmacol Ther 2009 47 570 578 19761716 10.5414/cpp47570

[B40] Wagg A Khullar V Marschall-Kehrel D Assessment of fesoterodine treatmetn in older people with overactive bladder: result of SOFIA, a double blind, placebo-controlled pan European trial Eur Urol Supplement 2011 10 276 277 [abstract]. e 2011

[B41] Chapple C Van KP Tubaro A Haag-Molkenteller C Forst HT Massow U Wang J Brodsky M Clinical efficacy, safety, and tolerability of once-daily fesoterodine in subjects with overactive bladder Eur Urol 2007 52 1204 1212 10.1016/j.eururo.2007.07.009 17651893

